# Reablement Services Across the World: Effectiveness and Impacting Factors

**DOI:** 10.1093/geroni/igab046.1611

**Published:** 2021-12-17

**Authors:** Stan Vluggen, Lise Buma, Barbara Resnick

## Abstract

Due to the ageing of the world population, solutions are necessary to reduce the increasing demand for care. Besides the need for more care, older people often wish to remain as independent as possible and retain as much control as possible. A possible solution are services based on the concept of reablement, which includes working in a more rehabilitative and person-centered manner and has been researched in various forms internationally. Reablement services are promising and use the patient's strengths and, through interdisciplinary cooperation, aims to achieve the goals important for, and set by, the individual to remain/become as independent as possible. During this symposium, five presenters from the US, New-Zealand, Norway and the Netherlands talk about the impact and implementation of reablement services. The first presentation is about the results of a systematic review of the effects of reablement on daily functioning and identifying common features of effective interventions. The second presentation is about a systematic scoping review mapping how physical activity strategies are integrated and explored in reablement research and identifying knowledge gaps. The third presentation is about the significant impact of COVID-19 and its associated restrictions on residents in assisted living communities. The fourth presentation is on combining lessons learned and practical implications from research on reablement services into the SELF-intervention. The fifth presentation describes the implications of funding on practice and outcomes of reablement. This symposium represents the current practice and future directions regarding implementation and research of reablement services across the world.

